# Dynamic near-field optical interaction between oscillating nanomechanical structures

**DOI:** 10.1038/srep10058

**Published:** 2015-05-27

**Authors:** Phillip Ahn, Xiang Chen, Zhen Zhang, Matthew Ford, Daniel Rosenmann, II Woong Jung, Cheng Sun, Oluwaseyi Balogun

**Affiliations:** 1 Mechanical Engineering Department, Northwestern University, Evanston, IL 60208; 2 Civil and Environmental Engineering Department, Northwestern University, Evanston, IL 60208; 3 Center for Nanoscale Materials, Argonne National Laboratory, Argonne, IL 60439-4806

## Abstract

Near-field optical techniques exploit light-matter interactions at small length scales for mechanical sensing and actuation of nanomechanical structures. Here, we study the optical interaction between two mechanical oscillators—a plasmonic nanofocusing probe-tip supported by a low frequency cantilever, and a high frequency nanomechanical resonator—and leverage their interaction for local detection of mechanical vibrations. The plasmonic nanofocusing probe provides a confined optical source to enhance the interaction between the two oscillators. Dynamic perturbation of the optical cavity between the probe-tip and the resonator leads to nonlinear modulation of the scattered light intensity at the sum and difference of their frequencies. This double-frequency demodulation scheme is explored to suppress unwanted background and to detect mechanical vibrations with a minimum detectable displacement sensitivity of 0.45 pm/Hz^1/2^, which is limited by shot noise and electrical noise. We explore the demodulation scheme for imaging the bending vibration mode shape of the resonator with a lateral spatial resolution of 20 nm. We also demonstrate the time-resolved aspect of the local optical interaction by recording the ring-down vibrations of the resonator at frequencies of up to 129 MHz. The near-field optical technique is promising for studying dynamic mechanical processes in individual nanostructures.

Low-dimensional nanostructures afford unprecedented control of mechanical^1^,^2^, optical^3^,^4^, electronic^5^, and thermal properties^6^ at the nanoscale. These nanostructures have inspired a variety of novel applications including nanoscale field-effect transistors^7^, nano-mechanical switches and mixers^8^, mechanical amplifiers^9^, opto-mechanical modulators^10^, bio-sensors^11^, and the observation of macroscopic quantum effects using nanomechanical oscillators with low thermal occupation numbers^12^. For sensing applications, nanoscale cantilever or doubly-clamped beams are typically employed. The shift in resonance frequency of the nanostructure is monitored and correlated with adsorption of binding analytes or perturbations by external forces. Detection of mechanical vibrations in these nanostructures with high sensitivity is of tremendous importance for enabling fundamental studies and applications that incorporate nanostructures as active elements.

Optical techniques are the most common approaches for detection of mechanical vibrations. These techniques are non-contact, non-destructive, and broadband. Optical techniques fall under two broad categories: interferometric and near-field methods. Interferometric methods^13^,^14^ are based on interference between a probe beam reflected from the vibrating structure and a reference beam, which converts the phase modulation of the probe beam to an intensity change. The displacement sensitivity of interferometric methods depends on the strength of the light-sample interaction. The minimum spot size of the probe beam in a classical interferometer is limited by far-field diffraction to about half the wavelength of the light source. Near-field optical techniques^10^,^15^,^16^, on the other hand, rely on the interaction between evanescent waves, e.g. from weakly guided modes in an optical fiber, and a vibrating nanomechanical structure in order to enhance the dynamic light scattering and displacement sensitivity.

Integration of evanescent optical wave techniques with scanning probe microscopy (SPM) has the potential to enable high sensitivity detection of nanomechanical vibrations and spatial imaging of their vibration mode shapes with nanoscale spatial resolution. However, since the intensity of evanescent light-matter interaction in SPM configurations^17^,^18^ is usually weak in comparison to the background, small amplitude vibrations can be difficult to detect. An optical nanofocusing approach that concentrates evanescent waves efficiently at the apex of an SPM probe can enhance the weak optical interaction with the vibrating structure and enable detection of mechanical vibrations on structures with sub-wavelength dimensions. In our previous work^19^, we reported a plasmonic nanofocusing approach that allows for local optical field confinement at the apex of an SPM tip (Fig. 1(a)). In our approach, free space light in the visible spectrum is coupled to surface plasmon polaritons (SPPs) on the shaft of a silver-coated SPM probe, while the tapered geometry of the shaft allows for adiabatic conversion of the SPPs to localized surface plasmons (LSPs) at the probe-tip. We demonstrated that the plasmonic nanofocusing probe serves as a local evanescent optical source and probe. Here, we explore the dynamic optical interaction between the probe tip which mechanically oscillates at a low frequency, and a high frequency nanomechanical resonator. We introduce a double-frequency demodulation approach to isolate the optical scattering between the probe-tip and sample by demodulating the optical signal recorded in the far-field at the difference frequency of the high and low frequency oscillators. Using this approach, we obtained a displacement sensitivity of 0.45 pm/Hz^1/2^, which is limited by shot noise and electrical noise in the detection electronics. We estimate the fundamental displacement noise floor of the approach in the absence of electronic noise to be 0.05 pm/Hz^1/2^, stemming from thermal vibrations in the low frequency SPM cantilever. The displacement sensitivity is better than that of evanescent optical transducers^10^,^20^ and SPM mechanical detection techniques^21^ by at least an order of magnitude, and it offers the prospect of imaging mechanical displacements with a lateral spatial resolution that is dictated by the spot size of the LSPs at the probe-tip, which is close to 20 nm. Furthermore, we explore the dynamic optical signal from the local probe-tip and sample interaction for time-resolved measurement of transient displacements of the nanomechanical resonator at multiple oscillation frequencies and for both horizontal and vertical vibration modes. The plasmonic nanofocusing approach provides a suitable platform for integrating mechanical and optical elements with the potential of reaching the ultimate thermal noise sensitivity limits of SPMs for displacement detection^22^.

## Methods

**Figure 1:**
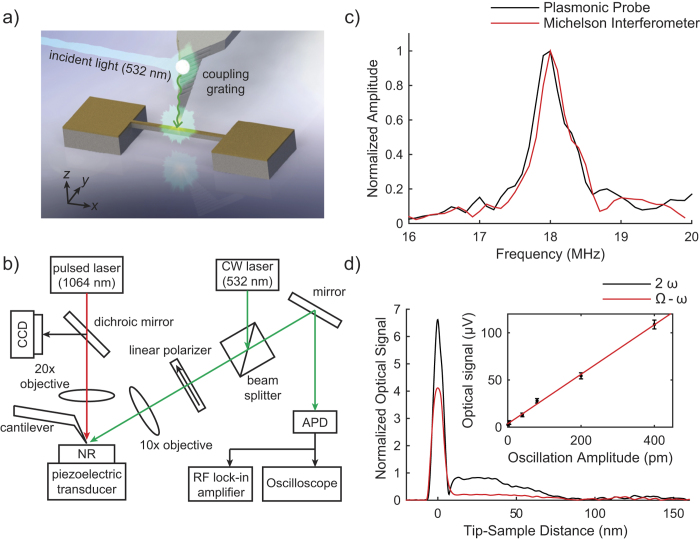
Local optical interaction between a plasmonic near-field optical probe and a nanomechanical resonator. (**a**) Schematic diagram of a low frequency atomic force microscopy (AFM) cantilever that supports the plasmonic nanofocusing probe. (**b**) Schematic diagram of experimental setup and optical detection electronics. The following labels are used, CW – continuous wave laser, RF – radio frequency, CCD – charge coupled device, and APD – avalanche photodetector. (**c**) Resonance spectrum of nanomechanical resonator measured by monitoring the modulated component of the probe-tip and sample interaction at Ω−*ω*, compared to a direct measurement of the sample surface displacement at Ω using an optical path-length stabilized Michelson interferometer. (**d**) Optical response of the plasmonic probe at frequencies, 2*ω* and Ω−*ω*, during the probe-tip and sample approach. *ω* = 2*πƒ*_*c*_ and Ω = 2*π*ƒ_s_, where ƒ_c_ and ƒ_s_ are the oscillation frequencies of the AFM cantilever and nanomechanical resonator. The probe-tip makes intermittent contact with the sample at *z*_*0*_ = 0. The inset in the figure shows the variation of the optical signal at Ω−*ω* with increasing vertical oscillation amplitude of the nanomechanical resonator.

Figure 1(b) shows a schematic of the experimental setup. The plasmonic nanofocusing probe is supported by an AFM cantilever with a resonant frequency of 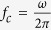 = 330 kHz. The cantilever has a spring constant of 54 N/m and a quality factor of 469. The AFM cantilever oscillates with a root mean squared (rms) amplitude of approximately 10 nm which is held constant during the experiment by the AFM controller feedback loop which adjusts the height (i.e. along the *z* direction in Fig. 1(a)) of a piezoelectric nano-positioning stage supporting the sample. A continuous wave *p*-polarized frequency-doubled neodymium yttrium aluminum garnet (Nd:YAG) laser with a wavelength of 532 nm is directed through a 10x microscope objective with a numerical aperture of 0.3, which focuses the light on a coupling grating on the edge of the AFM cantilever for excitation of LSPs. The peak power of the Nd:YAG laser that illuminates the coupling grating is less than 0.5 mW. Details of the coupling grating fabrication and probe-tip focused LSPs are discussed elsewhere^19^.

The nanomechanical resonators used in this study are doubly-clamped bilayer beams fabricated using top down nanofabrication techniques^14^. The dimensions of the resonator are as follows: length = 9.6–10.0 μm, width ~1.0 μm, and thickness ~250 nm comprised of silicon with thickness of 200 nm and a 50 nm thick chromium film. The metal coating on the resonator serves as a mirror to enhance the light scattering from the probe tip-sample interaction to the far-field. The scattered light is collected by the 10x microscope objective and directed to a high-speed avalanche photodetector (APD). An optical microscope that uses a white light source is positioned above the AFM cantilever for alignment of the 532 nm source onto the plasmonic coupling grating.

### Harmonic excitation

We use two actuation techniques in this study. In the harmonic excitation approach, the nanomechanical resonator is actuated by a 20 MHz longitudinal thickness-mode piezoelectric (PZT) transducer that sits on the nanopositioning stage, leading to out-of-plane bending of the resonator along the vertical axis. The voltage output from the APD is demodulated at the frequency of interest in a radio frequency lock-in amplifier (Stanford Research, SR844). For the double-frequency demodulation approach, the drive voltages to the AFM cantilever and the PZT actuator for the nanomechanical resonator are combined in an electronic mixer (Mini-Circuits, ZAD-1-1+) and the output signal at ƒ_s_ – ƒ_c_ is fed into the lock-in amplifier as the reference input.

### Photothermal excitation

In the photothermal excitation approach, a pulsed Nd:YAG laser (wavelength = 1064 nm, pulse width = 1 ns) is used to excite transient vibrations in the nanostructure. The pulsed laser is directed through a 20x microscope objective with a numerical aperture of 0.42 positioned above the AFM scanning stage, and it illuminates the surface of the nanomechanical resonator where the light is absorbed leading to impulsive heating, thermoelastic expansion of the chromium and silicon layers, and dynamic actuation of bending vibrations. The spot size of the pulsed laser is assumed to be close to the diffraction limit of 3 μm. For the transient measurements, the voltage output from the APD is digitized in an oscilloscope with a sampling rate of 5 gigasamples per second. The waveform is averaged 1000 times to suppress incoherent noise. The oscilloscope is triggered by sampling a portion of the pulsed laser source using a high-speed (1 GHz) silicon photodetector.

## Results and Discussion

### Near-field optical interaction

The resonator is harmonically driven at a frequency 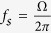 with a constant rms amplitude of less than 1 nm. The intensity of the local optical interaction depends on the length of the cavity *z*_*d*_ between the probe-tip and the nanomechanical resonator surface as expressed below^23^,
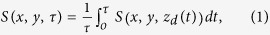

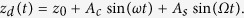


In the expressions, *A*_*c*_ and *A*_*s*_ are the oscillation amplitudes of the AFM cantilever and the nanomechanical resonator, *z*_*0*_ is the average cavity length, and *S*(*x,y,z*_*d*_(*t*)) is the instantaneous local optical signal, which depends on the cavity length and the lateral position of the probe-tip. The lock-in amplifier output, *S*(*x,y,τ*) is the modulated component of *S*(*x,y,z*_*d*_(*t*)) at the frequency of interest, obtained after integrating the signal with a time constant of τ. Figure 1(c) shows a comparison of the plasmonic probe optical signal *S*(*x,y,τ*) demodulated at 
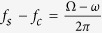
 and the optical signal obtained from similar experiment using a path-stabilized Michelson interferometer. In the Michelson interferometer measurement, the surface displacement of the resonator is monitored by tracking the modulated component of the probe light intensity at 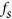 in the lock-in amplifier. The measured spectra are centered at 18 MHz, which correspond to the fundamental vertical flexural resonance frequency of the nanomechanical resonator.

### Suppression of background

Figure 1(d) shows a plot of the near-field optical signal demodulated at ƒ_s_ − ƒ_c_ and 2ƒ_s_ as a function of *z*_0_. The signal is enhanced at short distances between the probe-tip and nanomechanical resonator due to the local coupling of LSPs at the probe-tip to the nanomechanical resonator surface^24^,^25^. The signal decreases rapidly with distance, falling to approximately half of the maximum intensity level at a distance *z*_0 _~ 0.2*a*_*tip*_, where *a*_*tip*_ = 20 nm is the radius of curvature of the silver coated probe-tip^19^. The optical signal is compared to the response at 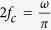 in the figure, which we have shown previously to suppress the contribution of background optical scattering from the resonator and the AFM cantilever. The background optical scattering varies slowly on the length scale of the resonator and AFM cantilever motion, leading to contributions to the optical signal at the fundamental frequencies ƒ_c_ and ƒ_c_ respectively. The optical signals in the figure exhibit similar trends for distances of less than 10 nm, however, at longer distances, the response at 2ƒ_c_ has a larger secondary lobe between 10 nm and 80 nm due to residual background scattering, which decays to the noise floor beyond this distance range.

The measurements suggest that the near-field optical interaction between the oscillating structures provides a strong vertical sensitivity due to the sharp drop in the optical signal as the probe-tip moves away from the nanomechanical resonator surface. To investigate this further, we plot the optical signal demodulated at 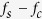 as a function of the rms oscillation amplitude of the nanomechanical resonator in the inset in Fig. 1(d). For the measurement, the oscillation amplitude of the AFM cantilever is held constant at approximately 10 nm in tapping mode, and the drive voltage to the PZT transducer for harmonic actuation of the nanomechanical resonator is varied. The intensity of the optical signal for each value of the resonator oscillation amplitude corresponds to the average of 1000 measurements, and the error bar is the standard deviation. The lock-in amplifier was set to average the optical signal with a bandwidth of 0.78 Hz. The measured optical signal increases linearly with the resonator amplitude over a range of 0.4 pm to 500 pm. The absolute amplitude of the nanomechanical resonator oscillation is calibrated with a Michelson interferometer. The slope of the linear fit in Fig. 1(d), ℜ = 0.26 μV/pm, represents the near-field optical responsivity of the probe-tip to the nanomechanical resonator displacement. At the smallest detectable displacement level in Fig. 1(d), which is 0.4 pm, the ratio of the measured optical signal to the standard deviation, which we define as the signal-to-noise ratio (SNR), is approximately 2.

### Near-field optical imaging of harmonic surface displacements

**Figure 2:**
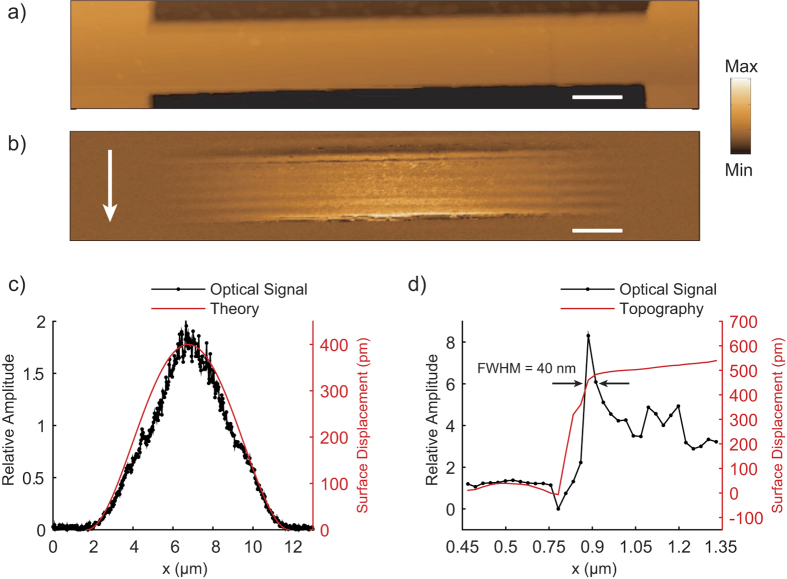
Near-field optical imaging of the probe-tip and sample interaction for a harmonically vibrating nanomechanical resonator. (**a**) Topography, (**b**) near-field optical image of the fundamental vertical bending mode shape of the nanomechanical resonator. The scale bars in the images are 1 μm long. (**c**) A line trace of the optical signal along the length of the nanomechanical resonator compared to a calculated mode shape of the fundamental bending mode using the Euler-Bernoulli model. (**d**) A line trace of the topography and optical signal close to an abrupt edge on the surface of the nanomechanical resonator.

To demonstrate the local nature of the plasmonic probe-sample interaction, we imaged the mode shape of the nanomechanical resonator by tracking the spatial variation of the optical signal at ƒ_s_ − ƒ_c_. Figures 2(a)–(d) compares pseudo color images of the topography and the optical signal. In the measurements, the sample is raster scanned from left to right relative to the probe-tip at a rate of 2 seconds per line. The probe-tip illumination direction is indicated by the arrow in Fig. 2(b). The optical signal is demodulated in the lock-in amplifier with a detection bandwidth of 0.78 kHz.

The near-field image follows the displacement profile for the fundamental mode shape of a doubly-clamped flexural beam, which has nodes at the fixed ends and an anti-node at the middle. The average of several line scans from the optical signal recorded along the nanomechanical resonator length is compared to the calculated mode shape for a doubly-clamped Euler-Bernoulli beam^26^ in Fig. 2(c), and the two profiles are in qualitative agreement. The absolute (rms) amplitude of the resonator displacement is calibrated with a Michelson interferometer. The fluctuations of the optical signal increase with displacement amplitude along the resonator and the signal decays to the noise floor of 5.9 pm close to the fixed ends. A lower vertical sensitivity is obtained in the lateral scanning measurement in comparison to the single-point measurement due to the large detection bandwidth of the measurement, which allows for signal averaging at each probe-tip position without spatially averaging the optical response as the sample is scanned.

### Lateral resolution

In addition, we estimate the lateral spatial resolution of the optical measurement by measuring the spatial width of the optical signal around an abrupt edge as the probe-tip scans across the width of the nanomechanical resonator. We assume that since the abrupt edge is smaller than the spot size of the probe-tip confined LSPs, it acts as a sub-wavelength optical scatterer. Figure 2(d) shows line profiles of the optical signal and the topography around the edge. The optical signal is enhanced as the probe-tip scans across the edge on the nanomechanical resonator surface due to strong diffraction of the incident light to SPPs and local scattering by the probe-tip to the far-field. This is observed in Fig. 2(b) as a bright white band close to one of the horizontal edges of the nanomechanical resonator. The full width at half maximum (FWHM) Δ*x* of the optical signal around the edge is 40 nm, from which we estimate the lateral spatial resolution of the measurement to be 0.5Δ*x = *20 nm^23^. The lateral spatial resolution is comparable to existing reports in the literature based on the apertureless near-field optical microscopy technique. However, the double-frequency demodulation approach used in this work provides high vertical sensitivity for measurement of small surface displacements at frequencies that are much higher than the AFM oscillation frequency. The fringes in the optical images are due to interference of SPPs excited by diffraction of the incident light at the sharp edges of the nanomechanical resonator, and SPPs coupled from the probe-tip to the resonator surface. An analytical model is presented in the Supplementary Information to describe these effects.

### Time-resolved detection of transient vibrations

**Figure 3:**
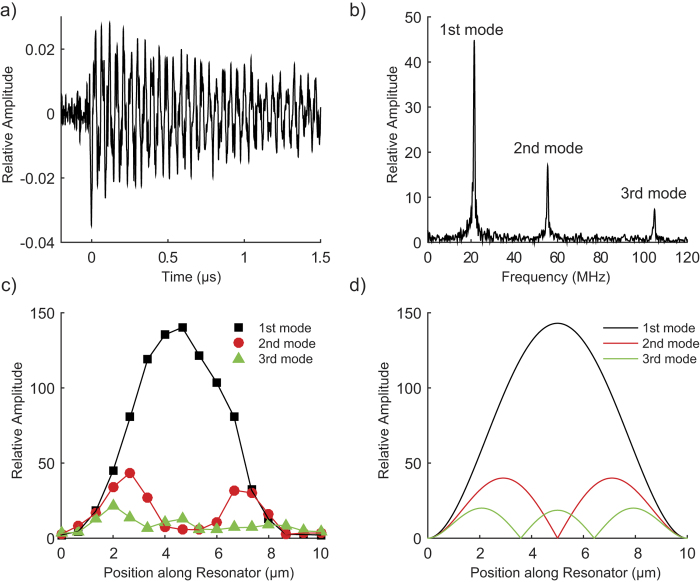
Local optical response of the probe-tip and sample interaction for an impulsively actuated nanomechanical resonator. (**a**) Transient optical signal showing the ring down vibrations of the nanomechanical resonator resulting from the pulsed photothermal heating, (**b**) amplitude spectrum of transient optical signal showing peaks at the fundamental frequency and harmonics of the vertical bending mode of the resonator, (**c**) a line trace of the amplitude peaks along the length of the nanomechanical resonator, and (**d**) calculated line profiles for the mode shape of the first three vertical bending modes from the Euler-Bernoulli model.

Next, we explore the broad detection bandwidth of the near-field optical interaction between the probe-tip and sample for tracking local transient surface displacements in the nanomechanical resonator. For the measurement, the resonator is actuated photothermally by the pulsed laser source. The PZT transducer used to excite harmonic displacements is turned off and the amplitude of the near-field optical signal at 2ƒ_c_ is monitored in the lock-in amplifier in order to maintain the coupling efficiency of the illumination source to the LSPs at the probe-tip. The pulsed laser source is positioned close to one of the fixed supports of the resonator and separated from the location of the plasmonic probe-tip and sample interaction by approximately 5.0 μm. Figure 3(a) shows the transient optical signal resulting from the probe-tip and sample interaction. The Fourier spectrum of the waveform shown in Fig. 3(b) shows three distinct amplitude peaks at 21, 55, and 105 MHz. To confirm that these frequencies correspond to flexural mode resonance frequencies, we compare them to the Euler-Bernoulli model for a doubly-clamped rectangular beam, which follows the relation,
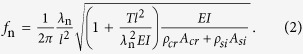


In the equation above, the symbols *ρ* and *A* represent the density and cross-sectional area and the subscripts *cr* and *si* denote the chromium and silicon layers. The effective flexural rigidity, *EI*, of the layered silicon-chromium beam depends on the elastic moduli of silicon *E*_*si*_ and chromium *E*_*cr*_, and the second moment of area *I*^27^. The residual tension along the length of the nanomechanical resonator is indicated by *T*, the beam length is *l*, and λ_*n*_ corresponds to the eigenvalue for flexural mode *n*. For the first three resonance modes, λ_*1*_ = 4.73, λ_*2*_ = 7.85, and λ_*3*_ = 11.0. Using the bulk values for the elastic properties and density (*E*_*si*_ = 169 GPa, *E*_*cr*_ = 140 GPa, *ρ*_*si*_ = 2.33 g/cm^3^, *ρ*_*cr*_ = 7.19 g/cm^3^) and the cross-sectional area of the resonator yields 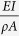 = 2.48 × 10^−7^ m^4^ s^−2^ for the vertical bending mode. The model fits the measured frequencies for a residual tension of 14 nN. We tracked the amplitude of these resonance frequencies with position along the nanomechanical resonator by scanning the sample stage relative to the plasmonic probe and pulsed laser source in discrete steps of 0.5 μm and recording the transient optical signal at each point. Figure 3(c) shows the amplitude profiles for the resonance modes between the fixed ends of the resonator. The amplitude profiles are in agreement with the expected mode shape for the first, second, and third flexural modes shown in Fig. 3(d) which are calculated using the Euler-Bernoulli model. The measured amplitude profiles have a slight asymmetry, which may be due to the thermal gradient along the resonator created by the photothermal source. The thermal gradient leads to a spatially varying thermal expansion along the long axis (or y axis) of the resonator.

The transient modulation of the optical signal measured in the pulsed photothermal actuation mode is not a result of the local near-field optical interaction alone. The measured intensity at the APD follows the relation,
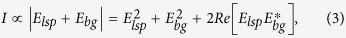
where *E*_*lsp*_ and *E*_*bg*_ are the electric fields of the scattered LSPs from the probe-tip sample interaction and the background light. In the harmonic displacement detection mode discussed in the previous section, we isolated the first term in Eqn. (3), i.e. the local optical probe-tip and sample interaction, by demodulating the measured intensity at the difference frequency ƒ_s_ − ƒ_c_ between the AFM cantilever and nanomechanical oscillations. In the transient case, the third term in Eqn. (3) amplifies the weak local optical field *E*_*lsp*_. When the probe-tip is retracted from the nanomechanical resonator surface by a distance of 1 μm, the amplitude of the ring down vibrations decrease to the noise floor suggesting the local origin of the displacement measurement^28^.

### Lateral flexural vibrations

**Figure 4:**
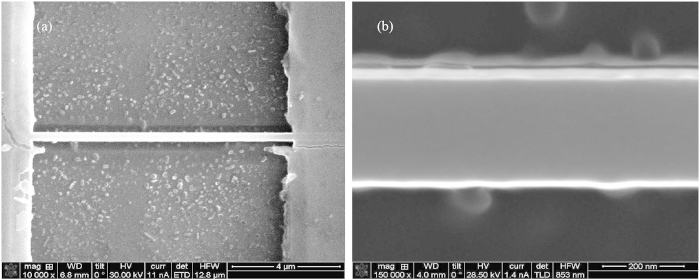
Focused ion beam milled nanomechanical resonator. Scanning electron microscopy images showing a panoramic view (**a**) and a close-up view (**b**) of a 270 nm wide nanomechanical resonator.

**Figure 5:**
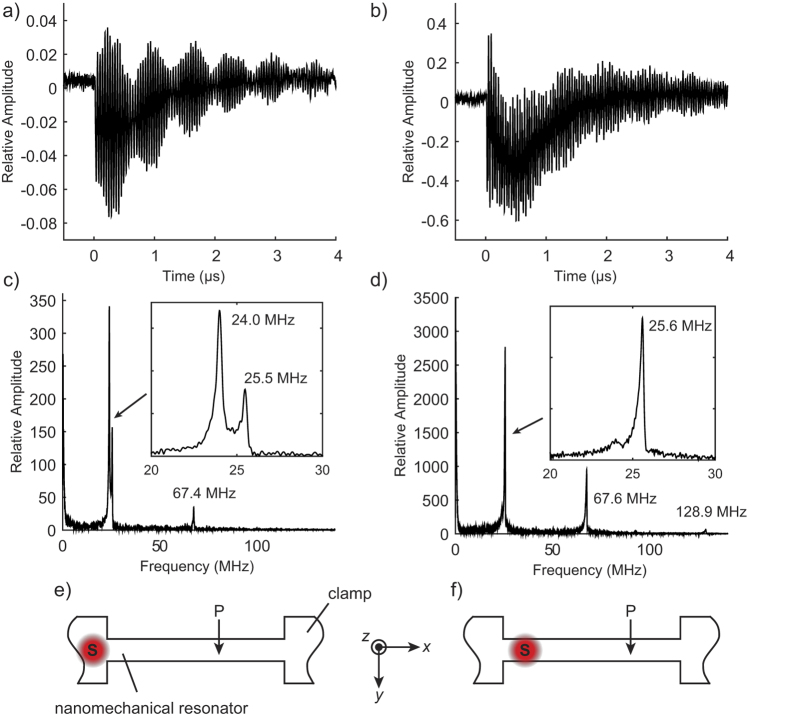
Local probe-tip and sample optical response to vertical and horizontal displacements of the nanomechanical resonator. (**a**) and (**c**) Optical signals showing the ring down vibrations of the nanomechanical resonator, and their corresponding amplitude spectrum (**b**) and (**d**), for two different configurations of the transient photothermal actuation of the nanomechanical resonator illustrated in (**e**) and (**f**). The following labels are used: S – photothermal source and P – plasmonic probe.

Finally, we show that optical interaction of the probe-tip and sample is also sensitive to in-plane (or horizontal) vibrations. For this experiment, the probe-tip is positioned close to a sharp edge on the nanomechanical resonator where strong diffraction of the incident probe light to SPPs amplifies the light scattered to the far-field as observed in Fig. 2(b). For this measurement, the width of the nanomechanical resonator is reduced to 270 nm which is close to the beam thickness (250 nm), in order to produce vertical and horizontal bending modes with close resonance frequencies. The width of the resonator is reduced from 1.0 μm by focused ion-beam milling using a 30 keV (48 pA) Ga^+^ ion source (FEI Nova 600 NanoLab). Scanning electron microscopy (SEM) images of the resulting nanomechanical resonator are provided in Fig. 4. Figure 5 compares the transient optical signals for two cases: (a) when the pulsed laser source is positioned on the clamp, and (b) when the laser is positioned directly on the nanomechanical resonator. For case (a), the transient optical signal shows a beating response due to interference of two oscillatory modes with close frequencies, 24.0 MHz and 25.5 MHz. These frequencies are evident in the amplitude spectrum of the waveform shown in Fig. 5(c). The quality factors of the resonance modes are 57 and 100. Using the Euler-Bernoulli model to match the theoretical and measured frequencies for the vertical and horizontal bending vibration modes, we obtained a residual tension of 8 μN, which is larger than the estimated value in the 1 μm beam. The horizontal resonance modes were not observed in the 1 μm nanomechanical resonator because they occurred at frequencies above 342 MHz. For case (b), the transient optical signal shows a slow amplitude relaxation lasting for 2 μs which is due to heat conduction into the substrate. Furthermore, the oscillatory response does not exhibit the characteristic beat phenomenon observed in case (a). Direct photothermal heating of the resonator leads to larger vibration amplitudes as compared to the clamp heating due to the increased bending moment resulting from differential thermal expansion of the chromium layer and the silicon beam^29^ and the compliance of the nanostructure. The amplitude spectrum of the optical signal for this case is shown in Fig. 5(d), where amplitude peaks at frequencies close to the fundamental horizontal resonance frequency and its harmonics are observed. The resonance frequencies of the horizontal modes increase slightly with beam heating, possibly due to elongation of the resonator which may result in a nonlinear mechanical response. The mechanism for detection of horizontal vibration is due to diffraction of SPPs confined around the edges of the nanomechanical resonator, such that the horizontal motion leads to redistribution of the light intensity recorded by the APD. As such, the sensitivity of this approach for detection of horizontal vibrations is highly dependent on the position of the probe-tip and sample interaction and the size of the aperture at the APD.

### Understanding the fundamental sensitivity limits

We investigated the limits to the displacement sensitivity of the near-field optical demodulation scheme by conducting a numerical Monte-Carlo simulation that incorporates the influence of various random noise sources on the optical probe-tip and sample interaction. We assume that the intensity of the LSPs scattered to the far-field from the probe-tip and sample interaction follows the quasi-static dipole approximation^17^,
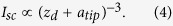


The output voltage 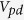 of the APD is modeled as a sum of the optical signal of interest 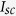, and noise sources including shot noise 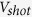 and dark current noise 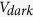,
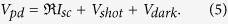


The first term in the equation is the signal arising from the LSPs scattered from the probe-sample interaction to the far field. This term includes the optical signal due to the harmonic displacements of the probe and sample, as well as stochastic thermal noise in the AFM cantilever. The DC component of the optical signal is filtered out by the lock-in amplifier and can be neglected in the calculation. Since it is impractical to simulate the detection bandwidth of 0.78 Hz used in the experiments presented in Fig. 1(d), the numerical simulations are performed with bandwidths of 10, 1, and 0.1 kHz. The calculations are performed 50 times for each value of the nanomechanical resonator displacement amplitude between 0.01 and 400 pm and the displacement amplitude of the AFM cantilever is fixed at 10 nm. Detailed formulas are included in the Supplementary Information.

**Figure 6:**
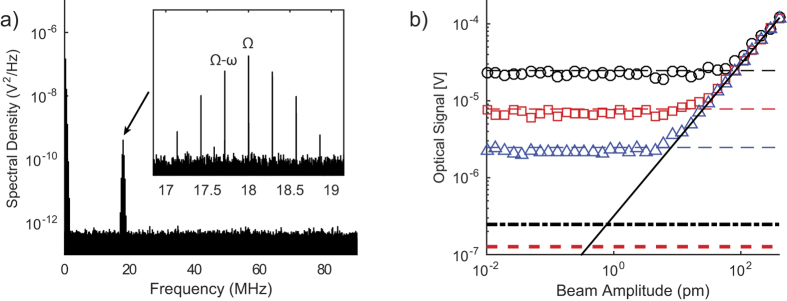
Monte Carlo numerical simulation of the displacement detection sensitivity of plasmonic nanofocusing probe. (**a**) Power spectral density of the simulated optical signal at the photodetector. Several harmonics of the AFM cantilever frequency, *ω*, are present along with a broad peak around the nanomechanical resonator frequency Ω. The inset shows several frequencies that arise due to beating between the nanomechanical resonator frequency and harmonics of the cantilever frequency. (**b**) Numerical calculation of the optical signal demodulated at the difference frequency, ƒ_s_ − ƒ_c_, as a function of sample oscillation amplitude. Simulations were performed with a bandwidth of 10 kHz (black circles), 1 kHz (red squares) and 0.1 kHz (blue triangles) along with the associated noise floor (dotted lines). The noise floor considering shot noise and dark-current noise at *B* = 1 *Hz* is shown with a dash-dot line, while shot noise alone is shown with a dashed line.

Figure 6(a) shows the power spectral density of 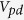 from a representative numerical simulation and the inset shows amplitude peaks at the resonance frequency of the nanomechanical resonator ƒ_s_, and sideband frequencies, ƒ_s_ − ƒ_c_ and ƒ_s_ + ƒ_c_ due to nonlinear optical mixing between the AFM cantilever and nanomechanical resonator oscillations. In Fig. 6(b), the simulated output voltage from the lock-in amplifier at ƒ_s_ − ƒ_c_ is plotted as a function of the displacement amplitude of the nanomechanical resonator for the three detection bandwidths. The noise floors corresponding to 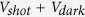 for each bandwidth are indicated by the horizontal dotted lines in the figure. The noise floor for the simulated bandwidths follows a simple relation 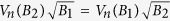, where *V*_n_(*B*_2_) and *V*_n_(*B*_1_) are the noise levels for detection bandwidths *B*_2_ and *B*_1_. Above the noise floor, the lock-in amplifier voltage increases linearly with displacement amplitude of the nanomechanical resonator as observed in the experiment. The solid line in the figure represents the sensitivity in the absence of the noise sources. Extrapolating the sensitivity line to a bandwidth of 1.0 Hz leads to a predicted noise floor of 0.78 pm/Hz^1/2^ and 0.40 pm/Hz^1/2^ at the dark current and shot noise limits, compared with our experimental result of 0.45 pm/Hz^1/2^. The noise floor considering only the thermal displacement noise in the probe cantilever is 0.05 pm/Hz^1/2^. The predicted noise levels may be overestimated due to the assumption that the cantilever thermal noise spectrum is flat in the numerical simulation. However, it is sufficient to conclude that the practical displacement sensitivity of the near-field optical probe is limited by the electronic and shot noise in the APD.

We were able to experimentally detect harmonic surface displacements on the order of 0.4 pm using a detection bandwidth of 0.78 Hz, which corresponds to a displacement sensitivity of 0.45 pm/Hz^1/2^. This sensitivity level is comparable to that of near-field optical transducers that rely on the perturbation of the optical cavity formed between an optical fiber containing weakly guided modes and a vibrating nanostructure. The advantage of our approach is that it provides high lateral spatial resolution through efficient confinement of LSPs at the probe-tip. Furthermore, there is room to improve the displacement sensitivity of our approach to the fundamental thermal noise limit with the use of a low noise optical detector and lower probe laser intensity levels in order to suppress the contribution from shot noise.

## Conclusion

In summary, we have studied the near-field optical interaction between two mechanically vibrating nanostructures: a plasmonic nanofocusing probe-tip and a high frequency nanomechanical resonator. The probe tip is supported by an AFM cantilever that oscillates at its fundamental resonance frequency. Taking advantage of light coupling to SPPs to enhance the electromagnetic field at the probe-tip and the tip-sample optical coupling, we showed that the intensity of light scattering to the far-field decreases rapidly as the probe-tip is withdrawn from the sample surface. We exploit the probe-tip sample interaction for detection of nanomechanical vibrations using a double-frequency demodulation scheme, where the optical signal is demodulated at the difference of the AFM cantilever and nanomechanical resonator oscillation frequencies. Using this approach, we obtained a displacement sensitivity of 0.45 pm/Hz^1/2^, which is limited by electronic and shot noise from the photodetector. In addition, the approach allows for detection of nanomechanical vibrations at frequencies much higher than the resonant frequency of the cantilever. Based on a Monte Carlo simulation that simulates various noise sources in our measurement system, we estimate the fundamental displacement sensitivity limit of the system to be 0.05 pm/Hz^1/2^ which is due to thermal vibration noise in the AFM cantilever. This sensitivity level is better than existing approaches based on evanescent optical fields by at least one order of magnitude. The lateral spatial resolution of the measurement approach, estimated from near-field optical images of the nanomechanical resonator is approximately 20 nm, which is comparable to the radius of the probe-tip. Furthermore, we demonstrated the broadband nature of the probe-tip sample interaction by tracking transient ring-down vibrations of the nanomechanical resonator. The amplitude spectrum of the recorded optical signals show multiple resonance frequencies up to 129 MHz, corresponding to the resonant frequencies of the vertical and horizontal vibration modes. This study provides the first experimental demonstration exploring near-field techniques in scanning probe microscopy for investigating dynamic mechanical processes in single nanoscale objects. In the future we envisage that the high spatial resolution and broad bandwidth enabled by the near-field optical interaction between the probe-tip and sample, can facilitate a wide range of applications including sensitive detection of single protein binding events^30^, investigations of elastic deformation of materials at the limit of continuum mechanics^31^, and determination of the mechanical properties of single metallic and semiconducting nanostructures based on measurements of the local interaction of elastic vibrations and electronic excitations^32^,^33^.

## Additional Information

**How to cite this article**: Ahn, P. *et al*. Dynamic near-field optical interaction between oscillating nanomechanical structures. *Sci. Rep.* **5**, 10058; doi: 10.1038/srep10058 (2015).

## Supplementary Material

Supplementary Information
