# Supplementary material for: Dynamic near-field optical interaction between oscillating nanomechanical structures
Source: Sci Rep. 2015 May 27;5:10058. doi: 10.1038/srep10058 (PMC4444852; doi:10.1038/srep10058)
Supplement: Supplementary Information [file srep10058-s1.pdf]

Supplementary Information

Dynamic near-field optical interaction between oscillating nanomechanical structures

Phillip Ahn[1#], Xiang Chen[1#], Zhen Zhang[1#], Matthew Ford[1#], Daniel Rosenmann[3], Il Woong Jung[3], Cheng Sun[1*], and Oluwaseyi Balogun[1,2*]

[1] Mechanical Engineering Department, Northwestern University, Evanston, IL 60208

[2] Civil and Environmental Engineering Department, Northwestern University, Evanston, IL 60208

[3] Center for Nanoscale Materials, Argonne National Laboratory, Argonne, IL 60439-4806

# These authors contributed equally to this work

* Please send all correspondence to Oluwaseyi Balogun o-balogun@northwestern.edu

Analytical model of intensity fringes

We adapt an analytical model presented in our previous work[1] to describe the fringes observed in the near-field optical image of the fundamental flexural vibration mode shape of the nanomechanical resonator shown in Fig. 2(b) and (d). The geometry of the problem is shown in Fig. S1(a). The nanomechanical resonator has the following dimensions: length = 9.6 μm, width ~ 1.0 μm, and thickness = 250 nm comprised of silicon with thickness of 200 nm and a 50 nm thick chromium film. In the analytical model, the total electric field at a given position of the probe-tip is given by

$$E_{tip}(y,z) = E_i(y,z) + E_{sp\_edge}(y,z) + E_{sp\_probe}, \qquad \text{(S1)}$$

where the subscripts *i, sp_edge* and *sp_probe* represents the projection of the illumination source along the nanomechanical resonator surface, SPPs excited by light diffraction at the edges of the resonator, and SPPs coupled to the resonator from the

plasmonic probe-tip. The complex electric field amplitudes of the three terms in Eqn. (S1) are,

$$E_i(y,z) = E_0,$$
$$E_{sp\_edge}(y,z) = E_1 e^{i(k_{sp,y}y - kycos\theta + k_{sp,z}z)},$$
$$E_{sp\_probe}(y,z) = E_2\sqrt{\frac{d_{probe}}{4\pi y}}\, e^{i(-2k_{sp,y}y + k_{sp,z}z)}, \quad \text{for y > 0.} \qquad \text{(S2)}$$

The time dependence, $e^{-i\omega t}$ is neglected for clarity and we consider only p-polarized (or transverse magnetic) waves. The constants E_0, E_1, and E_2 are real valued, k and k_{sp} are the magnitudes of the complex wave vectors of the illumination source and SPPs in the chromium film, and θ is the angle of the incidence with respect to the nanomechanical resonator surface. The components of k_{sp} along the y and z directions are denoted by $k_{sp,y}$ and $k_{sp,z}$, and k_{sp} is related to the light source frequency (ω) and the dielectric constant of air (ε_d) and the film (ε_m) by $k_{sp} = \frac{\omega}{c}\sqrt{\frac{\varepsilon_m \varepsilon_d}{\varepsilon_m + \varepsilon_d}}$, where c is the free space phase velocity of light. An additional phase term ($kycos\theta$) is presented in the complex amplitude of the resonator edge excited SPPs, to account for the phase lag between the excited SPPs and the illumination source. The length scale d_{probe} in Eqn. (S2) represents the optical scattering cross section of the probe-tip. The amplitude of the local evanescent electric field scattered by the probe-tip is approximated by $E_{nf}(y,z) \approx \alpha_{eff} E_{tip}(y,z)$,[2] where α_{eff} is the effective dipolar tip-sample polarizability. The total intensity of the optical field scattered to the far-field by the probe-tip is,

$$I_{nf} \propto \left|E_{tip}(y,0)\right|^2 = E_0^2 \left| \begin{matrix} 1 + \frac{E_1^2}{E_0^2} + \frac{E_2^2}{E_0^2}\frac{d_{probe}}{4\pi y} \\ + 2\frac{E_1}{E_0}\cos\left[\left(k_{sp,y} - kcos\theta\right)y\right] \\ + \frac{E_2}{E_0}\sqrt{\frac{d_{probe}}{\pi y}}\cos\left[2k_{sp,y}y\right] \\ +2\frac{E_1 E_2}{E_0^2}\sqrt{\frac{d_{probe}}{2y}}\cos\left[\left(3k_{sp,y} - kcos\theta\right)y\right] \end{matrix} \right| \qquad \text{(S3)}$$

The intensity of the scattered optical field has three terms that vary with the position variable y, and they result from the interference of: (1) the incident light and edge excited SPPs, leading to fringes with a wavelength of $\frac{2\pi}{k_{sp,y}-kcos\theta}$ = 2.63 μm, (2) the incident light and probe-tip excited SPPs, leading to fringes with a wavelength of $\frac{\pi}{k_{sp,y}}$ = 258 nm, and (3) the edge excited and probe-tip excited SPPs, leading to fringes with a wavelength of $\frac{2\pi}{3k_{sp,y}-kcos\theta}$ = 235 nm. The latter matches the measured fringe wavelength in the experiment. To obtain the calculated wavelengths, the SPP wavelength in the chromium film is assumed to follow the literature value of λ_{sp} = 516 nm for complex index of refraction of n = 2.91 +3.33i near the illumination source wavelength of 532 nm, and the real part of $k_{sp,y} = \frac{2\pi}{\lambda_{sp}}$. The angle of incidence of the illumination source with respect to the x-y plane of the nanomechanical resonator surface is varied to match the experiment, and estimated angle θ is 34°. Figure S1(b) shows a comparison of the analytical model and a line trace of the near-field optical image (Fig. 2(b)) along the width of the nanomechanical resonator. In the model, we only consider the contributions of the edge excited and probe-tip excited SPPs, by setting E_2 in Eqn. (S3) to zero, and we adjust the ratio of E_1 and E_2 as a free parameter to match the fringe profile in the experimental data. The two profiles are in good qualitative agreement.

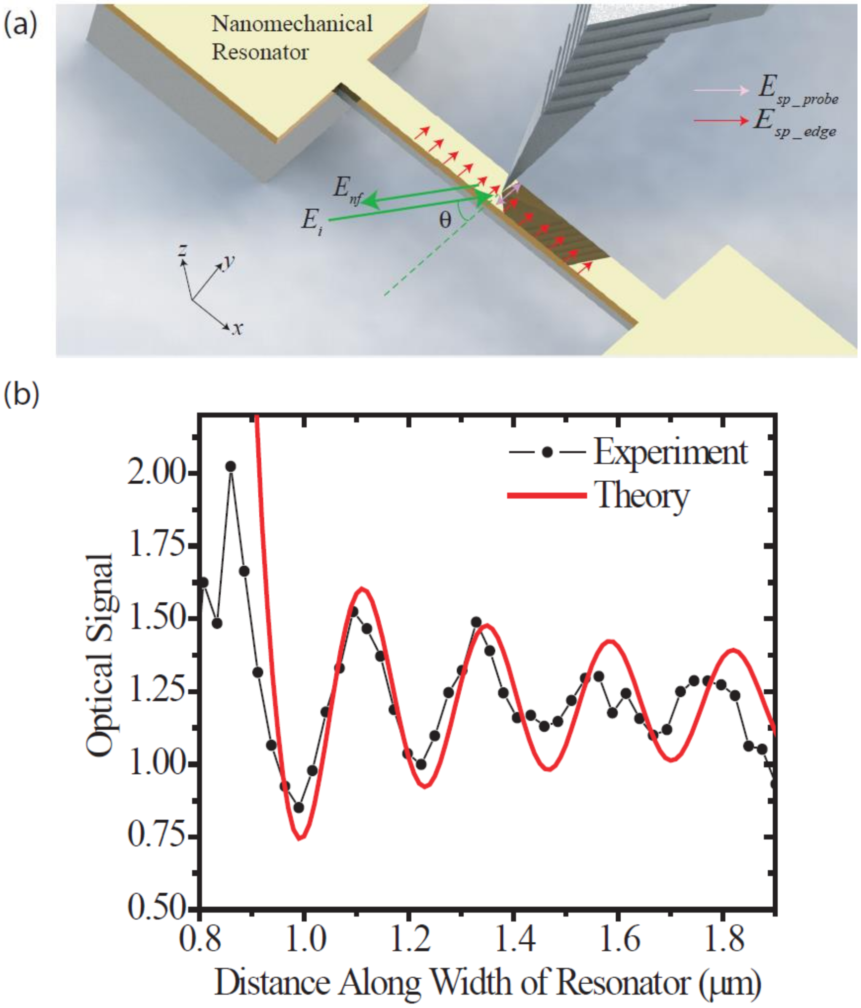

Figure S1. Modeling of surface plasmon polariton interference fringes on vibrating nanomechanical resonator. (a) Schematic illustration of the ray paths of various optical field components on the sample surface, and (b) a comparison of a line trace of the measured and calculated optical signals across the width of the nanomechanical resonator.

Scattering from surface defects. The orientation and wavelength of the SPP interference fringes depend on direction of the illumination source with respect to surface defects, such as cracks, on the nanomechanical resonator surface. The interference fringes modify the optical profile of the mode shape of the nanomechanical resonator in comparison to the expected mode shape. Figure S2 shows an illustrative example. The presence of a long crack in the chromium film leads to excitation of SPPs that interfere with the projection of the illumination source on the resonator surface. Figure S2(b) and (c) show

the topography and optical signal recorded on the nanomechanical resonator surface. Each data point in the image corresponds to the optical signal demodulated at the difference of the fundamental bending mode resonance frequencies of the AFM cantilever and the nanomechanical resonator. The optical image has two distinct features, namely, vertical interference fringes aligned along the x direction, and a mode shape that is non-symmetrical about the center of the nanomechanical resonator. Unlike the previous example, the fringes in the optical image result from interference of edge excited SPPs at the crack and the projection of the illumination source on the nanomechanical resonator surface. The edge excited SPPs propagate in the opposite direction to the projection of the illumination source on the chromium film as illustrated in the Fig. S2(a). We account for the counter-propagating electric fields terms by redefining the field terms as follows,

$$E_i(x,z) = E_0,$$
$$E_{sp\_edge}(x,z) = E_1 e^{i(k_{sp,y}x + kxcos\theta + \phi + k_{sp,z}z)}. \quad \text{(S4)}$$

The term ϕ in Eqn. (S4) is a constant phase offset. We sum up the field terms in Eqn. (S4) to obtain the intensity of the scattered optical field, following $I_{nf} \propto \left|E_i(y,0) + E_{sp\_edge}(y,0)\right|^2$, which yields a oscillatory term with a fringe wavelength of $\frac{2\pi}{k_{sp,x}+kcos\theta}$ = 286 nm, which is in agreement with the experimental data. Here, the real part of $k_{sp,x} = \frac{2\pi}{\lambda_{sp}}$. Figure S2(d) shows a comparison of the model prediction and a line trace of the optical signal along the resonator length. A line trace of the sample topography is also plotted in Fig. S2(d), and the region of abrupt height changes correspond to the location of the defects (cracks and contaminants) on the nanomechanical resonator surface. The model result in the figure is a product of I_{nf} and the expression for the fundamental mode shape of an Euler Bernoulli beam[3]. The ratio E_1/E_0 and the phase ϕ are used as free fitting parameters. The calculated and measured profiles for the optical signals are in good agreement. The optical profile of the flexural mode shape of the nanomechanical resonator is modified from the expected symmetrical shape due to the decaying oscillations on the chromium film.

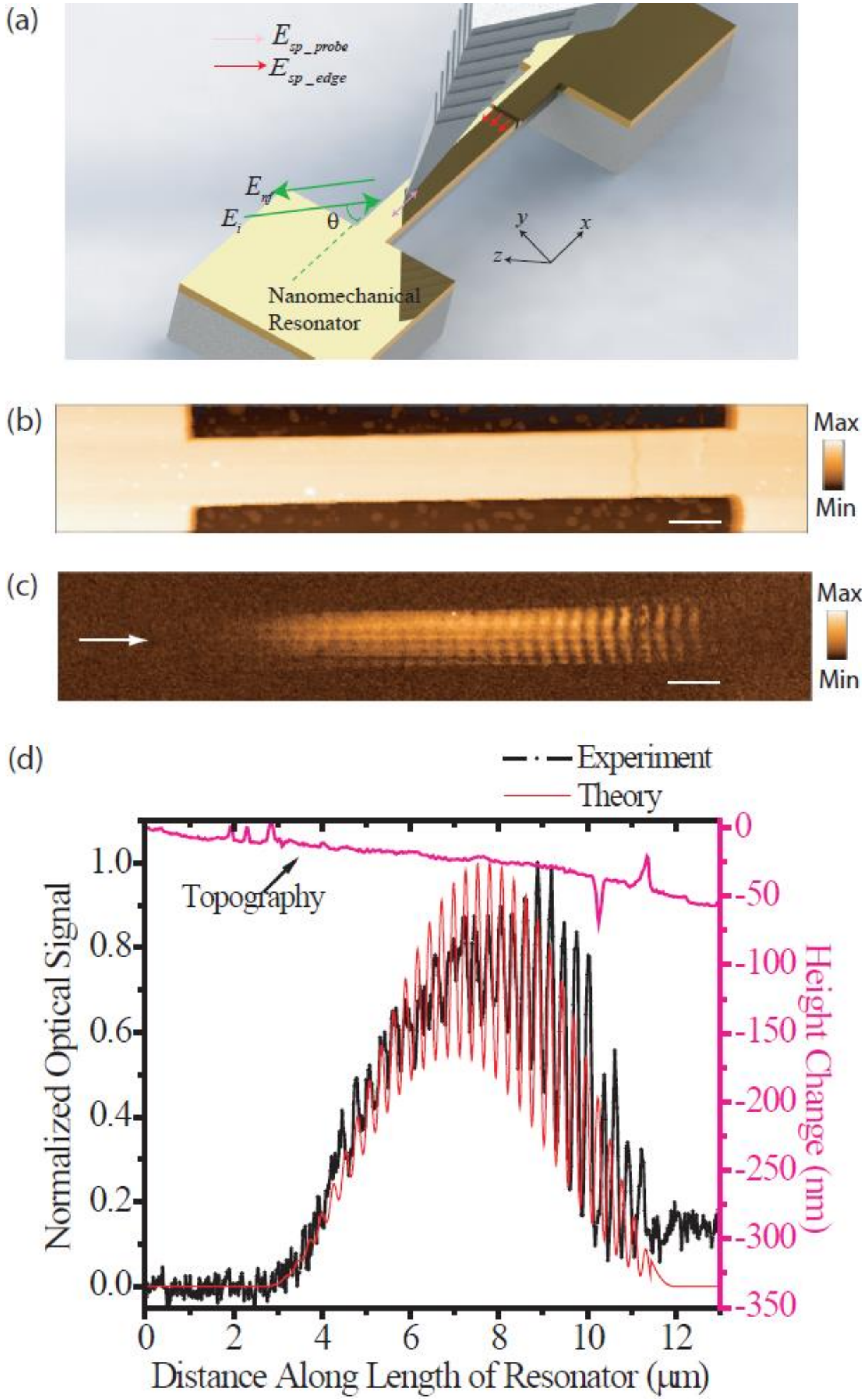
(a)
E_{sp_probe}
E_{sp_edge}
E_{rf}
E_i
θ
Nanomechanical Resonator
y
x
z
(b)
Max
Min
(c)
Max
Min
(d)
Experiment
Theory
Topography
Normalized Optical Signal
Height Change (nm)
Distance Along Length of Resonator (μm)

Figure S2. Near-field optical imaging of surface plasmonic polariton interference patterns on a vibrating nanomechanical resonator. (a) Schematic illustration of sample and probe-tip configuration, (b) topography, (c) near-field optical image demodulated at the difference frequency $\Omega - \omega$, where $\omega = 2\pi f_c$ and $\Omega = 2\pi f_s$, where f_c and f_s are the oscillation frequencies of the AFM cantilever and nanomechanical resonator. (d) Line profiles of the topography, and the measured and calculated optical signals along the length of the nanomechanical resonator for the fundamental vertical bending vibration mode. The scale bars in (b) and (c) are 1 μm long.

Monte-Carlo-Simulation

We implemented a numerical simulation in MATLAB in order to estimate the displacement sensitivity of our near-field detection scheme. A simulated discrete time series of the photodetector output voltage is generated according to the following equation

$$V_{pd}(t) = \Re I_{sc} + V_{shot} + V_{dark}. \quad \text{(S5)}$$

The sampling rate F_s is chosen such that $F_s/2 = 5f_s$, that is, the Nyquist frequency is five times the nanoresonator frequency. Each term in Eqn. (S5) is discussed in detail below. The sensitivity, $\Re$, is calibrated by comparing the simulated signal to the experiment for a harmonic beam displacement of 100 pm. The behavior of the lock-in amplifier is simulated by numerically demodulating the photodetector signal according to

$$V_{lock-in} = \frac{1}{T}\left[\left(\int_0^T V_{pd}(t)\sin(2\pi f_{ref}t)\ dt\right)^2 + \left(\int_0^T V_{pd}(t)\cos(2\pi f_{ref}t)\,dt\right)^2\right]^{1/2}, \quad \text{(S6)}$$

where $f_{ref} = f_s - f_c$. The first and second terms represent the in-phase and quadrature components of the APD output voltage at the reference frequency. The effective measurement bandwidth is $B = 1/T$, where T is the total duration of the time series. Numerical integration of the discrete time series is performed in MATLAB.

Intensity from LSPs scattered to the far field. The light intensity scattered by the probe-sample interaction, I_{sc}, is assumed to follow the quasi-static dipole approximation

$I_{sc} \propto \left(z_d + a_{tip}\right)^{-3}$. The probe-sample separation distance, z_d, includes the harmonic displacements of the probe and resonator, as well as thermal noise in the cantilever

$$z_d = z_0 + A_c \sin(2\pi f_c t) + A_s \sin(2\pi f_s t) + n_c(t). \tag{S6}$$

The thermal vibration noise of the un-driven AFM cantilever is measured using the optical lever detector in the AFM controller unit, and the noise power spectral density $PSD_{th}(\omega)$ of the measured data is integrated near f_c to obtain the mean squared thermal noise displacement, as described using the mathematical relation below,

$$\langle d_{th}^2 \rangle = \int_{F_{min}}^{F_{max}} PSD_{th}(\omega)\, d\omega, \tag{S7}$$

where $F_{max} = 2\pi f_c(1 + 1/2Q)$ and $F_{min} = 2\pi f_c(1 - 1/2Q)$, and Q = 469 is the quality factor of the AFM cantilever in air. The square root of Eqn. (S7) gives the approximate rms value of the thermal vibration noise near the fundamental mode, which is applied in Eqn. (S6) by adding Gaussian white noise, $n_c(t)$, with the same equivalent spectral density as the probe displacement near its fundamental mode. This is an overestimation of the actual displacement noise since in reality the noise spectral density is much lower off resonance. The thermal vibration noise of the nanomechanical resonator is assumed to be negligible since it is not detectable with the Michelson interferometer, operated at a sensitivity level of approximately 0.05 pm/Hz$^{1/2}$.

Shot noise and electrical noise. The shot noise contribution V_{shot} is modeled as Gaussian white noise with variance $\langle V_{shot}^2 \rangle = 2eB\Re_{APD}R_0P$, where e is the electronic charge, B is the measurement bandwidth, P is total optical power at the APD, $\Re_{APD}$ is the optical responsivity of the APD, and $R_0 = 50\,\Omega$, is the electrical resistance of the detection circuit. For a typical experiment, P = 1.3 μW. The dark-current noise in the APD (Thorlabs APD110A) and electrical noise in the lock-in amplifier (Stanford Research SR844) are modeled with Gaussian white noise according to the manufacturer's specifications.

REFERENCES

1 Zhang, Z., Ahn, P., Dong, B. Q., Balogun, O. & Sun, C. Quantitative Imaging of Rapidly Decaying Evanescent Fields Using Plasmonic Near-Field Scanning Optical Microscopy. *Sci. Rep.* **3**, (2013).

2 Huber, A. J., Ocelic, N. & Hillenbrand, R. Local excitation and interference of surface phonon polaritons studied by near-field infrared microscopy. *Journal of Microscopy-Oxford* **229**, 389-395, (2008).

3 Meirovitch, L. *Fundamentals of Vibrations*. (McGraw-Hill Higher Education, 2001).
